# Citizen science shows systematic changes in the temperature difference between air and inland waters with global warming

**DOI:** 10.1038/srep43890

**Published:** 2017-03-06

**Authors:** Gesa A. Weyhenmeyer, Murray Mackay, Jason D. Stockwell, Wim Thiery, Hans-Peter Grossart, Pétala B. Augusto-Silva, Helen M. Baulch, Elvira de Eyto, Josef Hejzlar, Külli Kangur, Georgiy Kirillin, Don C. Pierson, James A. Rusak, Steven Sadro, R. Iestyn Woolway

**Affiliations:** 1 Department of Ecology and Genetics/Limnology, Uppsala University, Norbyvägen 18D, 752 36 Uppsala, Sweden; 2 Science and Technology Branch, Environment and Climate Change Canada, 4905 Dufferin Str. Toronto, Ontario, M3H5T4, Canada; 3 Rubenstein Ecosystem Science Laboratory, University of Vermont, 3 College Street, Burlington, Vermont 05401, USA; 4 Institute for Atmospheric and Climate Science, ETH Zurich, Universitätstrasse 16, 8092 Zürich, Switzerland; 5 Department of Hydrology and Hydraulic Engineering, Vrije Universiteit Brussels, Pleinlaan 2, 1050 Brussels, Belgium; 6 Department of Earth and Environmental Sciences, KU Leuven, Celestijnenlaan 200E, 3001 Leuven, Belgium; 7 Department Experimental Limnology, Leibniz Institute of Freshwater Ecology and Inland Fisheries, Alte Fischerhuette 2, 16775 Stechlin, Germany; 8 Institute for Biochemistry and Biology, Potsdam University, Maulbeerallee 2, 14469 Potsdam, Germany; 9 Remote Sensing Department, National Institute of Space Research (INPE), São José dos Campos, São Paulo, Brazil; 10 School of Environment and Sustainability and Global Institute for Water Security, University of Saskatchewan, 11 Innovation Boulevard, Saskatoon, SK S7N 3H5, Canada; 11 Marine Institute, Furnce, Newport, Co. Mayo, Ireland; 12 Biology Centre CAS, Institute of Hydrobiology, Na Sádkách 7, 370 05 České Budějovice, Czech Republic; 13 Centre for Limnology, Institute of Agricultural and Environmental Sciences, Estonian University of Life Sciences, 61117 Rannu, Estonia; 14 Dept. of Ecohydrology, Leibniz Institute of Freshwater Ecology and Inland Fisheries, Müggelseedamm 310, 12587 Berlin, Germany; 15 Dorset Environmental Science Centre, Ontario Ministry of the Environment and Climate Change, P0A 1E0, Dorset, ON, Canada; 16 Department of Biology, Queen’s University, K7L 3N6, Kingston, Canada; 17 Department of Environmental Science and Policy, University of California, Davis, CA 95616, USA; 18 Department of Meteorology, University of Reading, Reading, UK

## Abstract

Citizen science projects have a long history in ecological studies. The research usefulness of such projects is dependent on applying simple and standardized methods. Here, we conducted a citizen science project that involved more than 3500 Swedish high school students to examine the temperature difference between surface water and the overlying air (T_w_-T_a_) as a proxy for sensible heat flux (Q_H_). If Q_H_ is directed upward, corresponding to positive T_w_-T_a_, it can enhance CO_2_ and CH_4_ emissions from inland waters, thereby contributing to increased greenhouse gas concentrations in the atmosphere. The students found mostly negative T_w_-T_a_ across small ponds, lakes, streams/rivers and the sea shore (i.e. downward Q_H_), with T_w_-T_a_ becoming increasingly negative with increasing T_a_. Further examination of T_w_-T_a_ using high-frequency temperature data from inland waters across the globe confirmed that T_w_-T_a_ is linearly related to T_a_. Using the longest available high-frequency temperature time series from Lake Erken, Sweden, we found a rapid increase in the occasions of negative T_w_-T_a_ with increasing annual mean T_a_ since 1989. From these results, we can expect that ongoing and projected global warming will result in increasingly negative T_w_-T_a_, thereby reducing CO_2_ and CH_4_ transfer velocities from inland waters into the atmosphere.

Research organizations and funding agencies are increasingly striving to include society in science, to justify the use of public funds for research and to raise societal awareness and scientific knowledge. In the best cases, citizen participation is a win-win situation where society becomes more informed and scientists secure valuable data^1^,^2^. To develop reliable citizen science projects is a challenge because they require simple and unambiguous descriptions and methods that are standardized and easy to apply^3^,^4^. Here, we used citizen science to better understand temporal and spatial variation in the potential transfer of the greenhouse gases carbon dioxide (CO_2_) and methane (CH_4_) from inland waters to the atmosphere by engaging secondary school students to collect water and air temperature data from a diverse set of water bodies across a large geographical region.

## Theory behind the citizen science project

The transfer of CO_2_ and CH_4_ from inland waters to the atmosphere is an important component of the global carbon cycle^5^. Recent estimates demonstrate that about 2.1 PgC yr^−1^ are emitted from inland waters to the atmosphere in the form of CO_2_^6^, an amount comparable to CO_2_ uptake by oceans (~2.0 PgC yr^−1^)^7^. The emission estimates, however, are still uncertain because they are based on a simplified measure of the gas transfer velocity *k* where the sensible heat flux (Q_H_) has been neglected^6^. Although Q_H_ is a relatively minor component of the total heat flux^8^,^9^, Q_H_ can substantially enhance *k* and thereby the total emission flux^10^,^11^,^12^,^13^,^14^. For example, measured and calculated CO_2_ emission flux from a lake can differ by up to 79% when Q_H_ is not considered in the calculation of the emission flux^15^.

Q_H_ enhances *k* when it is directed upward, i.e. when heat is transferred from water to the air which occurs when water is warmer than the overlying air. Under these conditions the buoyancy flux is negative and the resulting turbulent mixing from heat loss is responsible for the majority of gas exchange^14^. Thus, the temperature difference between water and air (T_w_-T_a_) is an important measure because its sign regulates whether Q_H_ is directed upward, i.e. from water into the atmosphere, or downward, i.e. from the atmosphere into water according to^10^:


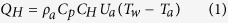


where *ρ*_*a*_ is the density of air, *C*_*p*_ is the specific heat capacity of air at constant pressure (1005 J kg^−1^ K^−1^), *C*_*H*_ is the turbulent transfer coefficient for sensible heat, and *U*_*a*_ is the near-surface wind speed. Although turbulence from heat loss is known to be the primary driver of gas flux in many lakes around the world^16^,^17^, the distribution, sign, and magnitude of T_w_-T_a_ are still unknown for most of our inland waters. Thus, the precision of present greenhouse gas emission estimates from inland waters remains unclear.

The distribution, sign, and magnitude of T_w_-T_a_ vary across water bodies and over time. We aimed to fill the present knowledge gap on spatial T_w_-T_a_ variation using data from citizen scientists. To fill the present knowledge gap on temporal T_w_-T_a_ variability, we used high-frequency temperature data from 14 lakes distributed around the globe. In addition to the assessment of T_w_-T_a_ spatial and temporal variability, we also set out to find a relationship between T_w_-T_a_ and the color of water. Water color affects the attenuation of solar radiation^18^,^19^ which most likely results in changes to T_w_-T_a_. The possible effects of water color on T_w_-T_a_ are highly relevant because waters in the Northern Hemisphere are becoming browner^20^,^21^. As a final step, we analyzed the inter-annual development of T_w_-T_a_ using the longest available time series of high-frequency water and air temperature measurements from 1989 to 2015.

## Methods

### Design of the citizen science project

For the citizen science project, we chose high school students between 14 and 16 years of age because these students already have a basic knowledge in natural sciences and soon have to decide if they would like to continue studying this discipline. We introduced the citizen science project via webpages, Facebook and other social media, and produced a short video (http://www.teknat.uu.se/bruntvatten/). Originally, we intended to involve 100 classes across Sweden but due to unexpected high interest already during the first hours of registration, we extended participation to 240 classes. We sent packages to all the schools, each of them containing field protocols, a thermometer, sampling tubes, and a detailed experimental description for teachers. In addition, the pack contained a detailed questionnaire that students could choose to fill out, which had a direct connection to the teaching goals of grades 7 to 9 in natural sciences.

We asked the teachers and students to choose a water body near their school and to measure surface water temperature at 0.5 m water depth and air temperature at 1.5 m above the sampling site. At each sampling site, the students were asked to fill in a protocol with weather observations, their temperature measurements, pH measurements, their exact sampling location and an estimation of water color using the Forel-Ule color index scale^22^. The students were also asked to take a photograph of their water body and to fill 50-ml sampling tubes with water from 0.5 m water depth from each measuring site, which they could then send to the limnological laboratory at Uppsala University. The water samples were used as a control for the students’ water color estimates. We chose 10 of the most brownish, 10 of intermediate brownish, and 10 of the most transparent water samples, as indicated by photographic records taken at each site by the students, and measured the absorbance at 420 nm in a 1 cm cuvette (Abs_420nm/1cm_) as a proxy of water color.

The project began in May 2016 when school teachers registered. The actual sampling took place between August 15 and September 30 in 2016. At the beginning of September, school classes were invited to have a 15-minute conversation with the project leader via Skype. Over the course of the project we constantly provided feedback to teachers and students, and at the end of the project we sent out a project evaluation sheet to all the class teachers.

### High-frequency temperature measurements

We combined the temperature data from the citizen science project with high-frequency temperature measurements in inland waters at about 0.5 m water depth and the overlying air at about 1.5 m above the water in 14 diverse lakes from four continents and ten countries (Table 1). The high-frequency temperature data are available via the GLEON network at http://www.gleon.org. We included complete time series of high-frequency temperature data during the open water season from May to October. All lakes had at least one year of complete data, except for the Brazilian floodplain lake for which only 14 days of data were available. The longest high-frequency time series with day and night and under ice water temperature measurements was available from Lake Erken, Sweden (Table 1). This time series was used to analyze inter-annual variation of T_w_-T_a_ from 1989 to 2015. Four years, i.e. 1993, 1994, 1996, and 2003, were not considered in this study since they lacked more than 2900 out of 8760 measurements (or 8784 during a leap year) due to instrumentation failure.

### Statistics

All statistical tests were run in JMP, version 12.0. Due to non-normally distributed data, tested by a Shapiro-Wilk test, we used the non-parametric Wilcoxon test for group comparisons and the non-parametric Mann-Kendall test for trend analyses. The Mann-Kendall test^23^ tests if long-term changes of a variable are significant (*p* < 0.05). For the trend analyses we used yearly median values. We also calculated the Theil-slope to get a quantitative measure of changes over time.

## Results and Discussion

### Spatial variation in T_w_-T_a_ and the influence of water color – data from the citizen scientists

**Figure 1:**
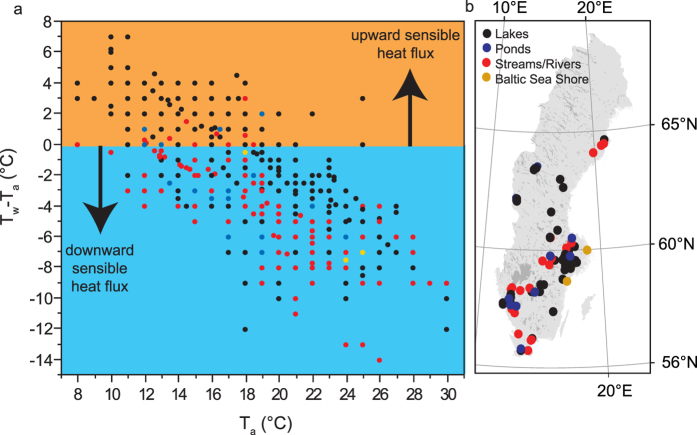
Relationship between air temperature (T_a_) and the temperature difference between surface water and the overlying air (T_w_-T_a_). All temperatures (1355 paired air and water temperatures) were reported from high school students and measured in 11 small ponds, 49 lakes, 22 streams/rivers and at 2 Baltic Sea shore sampling sites across Sweden (panel b) between August 15 and September 30, 2016. The relationship is linear and significant (panel a; *R*^*2*^ = 0.54, *p* < 0.0001). The orange color indicates an upward sensible heat flux and the blue color when there is a downward sensible heat flux. The Swedish map was created in ARC GIS, version 10.3.1., using a shape file with open data obtained from the Swedish Meteorological and Hydrological Institute (http://www.smhi.se) under the agreement of the licensing terms specified in Creative Commons Attribution 4.0. (https://creativecommons.org/licenses/by/4.0/) The dots in the map and the text were finally modified in Adobe Illustrator version CS6.

We received protocols and samples back from 80% (192) of the 240 registered classes. A number of teachers were unable to complete the project because they had changed jobs or were on parental or sick leave. In total, we received 1355 paired water (T_w_) and air (T_a_) temperatures. The measurements were recorded in 11 small ponds, 49 lakes, 22 streams/rivers and at two Baltic Sea shore sampling sites between August 15 and September 30. Most sampling sites were located in Sweden’s most populated areas such as Stockholm, Malmö and Gothenburg but the distribution of sites was surprisingly extensive across Sweden, spanning 56 to 65°N (Fig. 1b). The distribution of sites clearly showed a general interest from schools in participating in research projects, not only in cities that host Universities.

Across all water bodies sampled by the students, T_a_ varied between 6 and 30 °C (median T_a_: 19 °C), and T_w_ varied between 6 and 28 °C (median T_w_: 16 °C). Despite a similar T_a_ and T_w_ range, T_w_-T_a_ showed substantial variations, ranging between −14 and 7 °C, with a median of −2 °C. The negative median T_w_-T_a_ implies that a majority of the Swedish waters exhibited a downward sensitive heat flux, i.e. from air to water during the day between August to September around the September equinox (Fig. 1), suggesting a suppression of the gas transfer velocity *k*. T_w_-T_a_ was significantly different between standing (ponds and lakes) and running waters (streams and rivers; non-parametric Wilcoxon test: *p* < 0.001). This result was expected because streams have higher water turbulence without thermal stratification which strongly influences the heat exchange between water and the overlying air.

We found no relationship between T_w_-T_a_ and the color of water with data from citizen scientists, probably because of two reasons. First, water color estimates from the citizen scientists were not reliable when we calibrated the students’ values against Abs_420nm/1cm_ measurements, suggesting that the Forel-Ule color index scale is too subjective, at least when many individual participants are involved. Second, we did not find a relationship between Abs_420nm/1cm_ and T_w_-T_a_ despite Abs_420nm/1cm_ ranging between 0.001 and 0.4 and T_w_-T_a_ between −7 °C and 5 °C using the 30 water samples for which we determined Abs_420nm/1cm_ (*R*^*2*^ = 0.001, *p* > 0.05). Thus, our results suggest that the influence of water color on spatial T_w_-T_a_ variability is negligible.

### Temporal variation in T_w_-T_a_ – data from high frequency measurements

**Figure 2:**
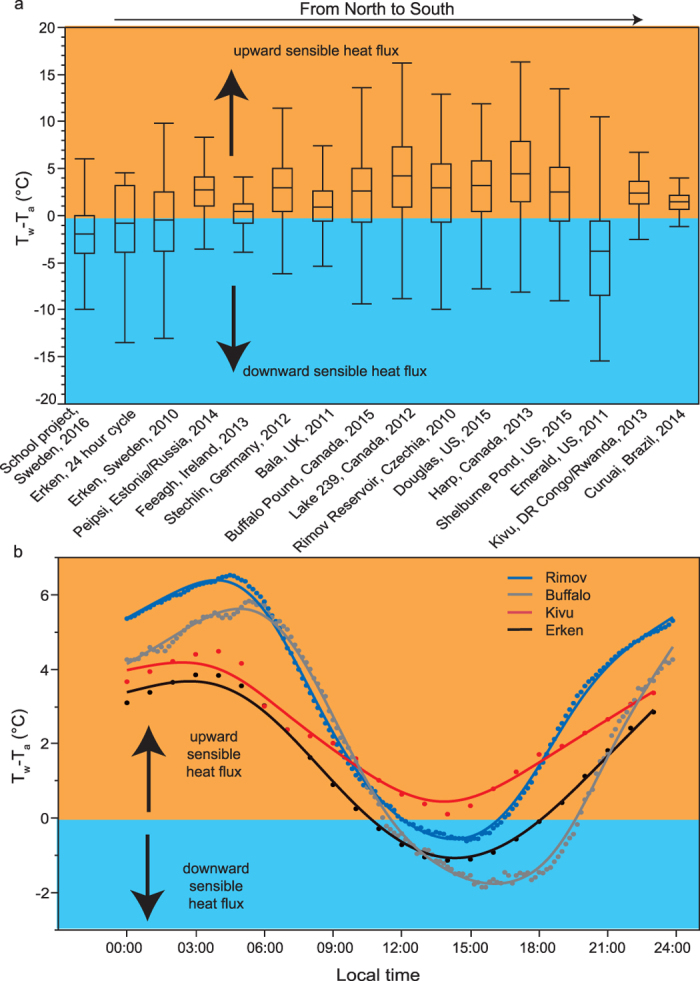
Spatial and temporal variations in the temperature gradient between surface water and the overlying air (T_w_-T_a_). Panel a: box plots of T_w_-T_a_ variations across small ponds, lakes, streams/rivers and the shoreline of the Baltic Sea (school project, *n* = 1355), within 24 hours on September 1, 2014 in the Swedish Lake Erken (*n* = 24), and within a year during the open water season May to October, based on high-frequency temperature measurements from 14 lakes across the globe (Table 1). Box size corresponds to the interquartile range and whiskers to a distance of 1.5 times the interquartile range from the 25th and 75th quantile, respectively. When more than one year of data was available we chose the time period 2010–2015 and plotted the year with the highest T_w_-T_a_ variance. Panel b: T_w_-T_a_ variations within 24 hours in the most northern and dimictic lake (Erken), a tropical lake (Kivu), a shallow polymictic lake (Buffalo) and a deep small reservoir (Rimov). The T_w_-T_a_ values are median values during May to October from all available years (Table 1). Data points from each lake are connected by a spline function with lambda equal to 0.05. In both panels the orange color indicates when there is an upward sensible heat flux and the blue color when there is a downward sensible heat flux.

We found that the geographical variation in T_w_-T_a_ from the citizen science project was similar to the intra-annual variation in T_w_-T_a_ of individual lakes (Fig. 2a). The intra-annual T_w_-T_a_ variability was even covered within a single 24-hour period at Lake Erken (second box plot in Fig. 2a). Thus, a very strong diurnal forcing on T_w_-T_a_ exists and is comparable between small and large, shallow and deep, as well as polymictic and dimictic lakes (Fig. 2b). Within 24-hour periods, a shift between downward and upward sensible heat flux is evident for the majority of lakes where the upward heat flux dominates during the 15 hours between 20:00 and 11:00 local time and the downward heat flux dominates during the day between 11:00 and 16:00 local time (Fig. 2b). Our results suggest that the gas transfer velocity *k* is generally highest around 04:00 during early morning and lowest during early afternoon. The associated large diurnal variations in Q_H_, and in particular the daily shift from upward to downward Q_H_, need to be taken into consideration for *in-situ* gas emission measurements. At present, gas emission estimates that neglect Q_H_ or that are based only on daytime measurements when the gas transfer is suppressed by a downward Q_H_ are lower than actual gas emissions from inland waters.

On an annual basis (in this study equal to the open water season May to October), we commonly found a net upward sensible heat flux in the 14 diverse lakes. The only clear exception was a small high-altitude lake which showed an annual net downward sensible heat flux (Fig. 2a). Even the most northern lakes, i.e. Lake Erken and Lough Feeagh, occasionally shift from having an annual net downward sensible heat flux to having an annual net upward sensible heat flux. We interpret geographical T_w_-T_a_ differences, in particular T_w_-T_a_ differences between high altitude/latitude and tropical lakes as a result of higher solar radiation during the open water season May to October, which is well known to influence the sensible heat flux^24^.

### Inter-annual variation of T_w_-T_a_

**Figure 3:**
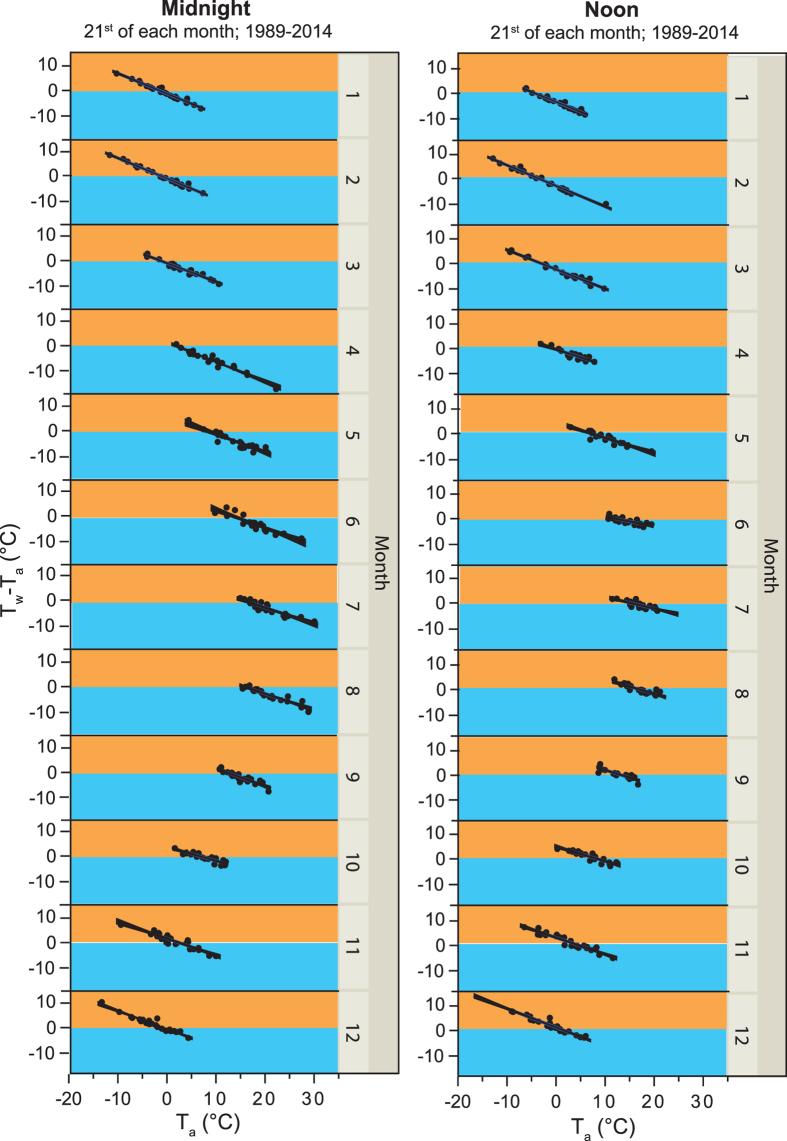
Increasing difference between surface water and air temperature (T_w_-T_a_) with increasing air temperature (T_a_) in Lake Erken. Shown are year-to-year variations during 1989 to 2015 of *in situ* T_a_ and T_w_-T_a_ from Lake Erken at day 21 of each month at midnight (left panel) and noon (right panel). All relationships are linear and highly significant (*p* < 0.0001). The orange color indicates when there is an upward sensible heat flux and the blue color when there is a downward sensible heat flux.

We found a significant decreasing trend over time in yearly median T_w_-T_a_ in Lake Erken during 1989 to 2015 (Mann-Kendall trend test: *p* < 0.01). Since 1989 T_w_-T_a_ has, on average, decreased by 0.07 °C yr^−1^. Over the same time period, T_a_ has significantly increased by, on average, 0.08 °C yr^−1^ (*p* < 0.05). On further examination of T_w_-T_a_, we found that the temperature difference was strongly negatively related to T_a_, both across Sweden (Fig. 1) and on an inter-annual scale (Fig. 3). The relationship was remarkably linear. To rule out autocorrelation we examined the relationship from a lake surface energy balance perspective. Under equilibrium conditions, a balance exists between net radiation (*R*_*N*_) and total turbulent heat exchange with the atmosphere:


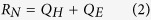


where Q_E_ is latent heat flux. What constitutes the surface where these fluxes take place is left ambiguous in this approximate analysis, but is assumed to be thick enough to absorb most of the penetrating shortwave radiation and to minimize temperature fluctuations. *Q*_*E*_can be expressed in a manner analogous to (1) but here we choose to follow a Bowen ratio approach. The Bowen ratio, defined as


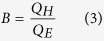


can be approximated as^25^


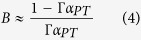


In this expression *α*_*PT*_ is a constant generally set to 1.26 for evaporation over saturated surfaces^26^, and Γ arises from the Penman combination approach to evaporation^27^. It is defined as


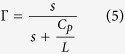


where *L* is the latent heat of vaporization (2.5 × 10^6^ J kg^−1^) and *s* is the slope of the saturation specific humidity curve. The Penman combination approach, and the Priestley – Taylor simplification have been used successfully in countless meteorological studies for many years, including studies over bodies of water^28^,^29^,^30^. Their success arises because Γ scales nearly linear with respect to air temperature, and if evaluated at the mean temperature 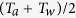 leads to only small errors in estimates of evaporation, even when T_w_-T_a_ is relatively large^25^. Combining (1), (3), and (4) we find that (2) can be written as:


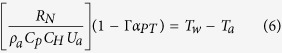


**Figure 4:**
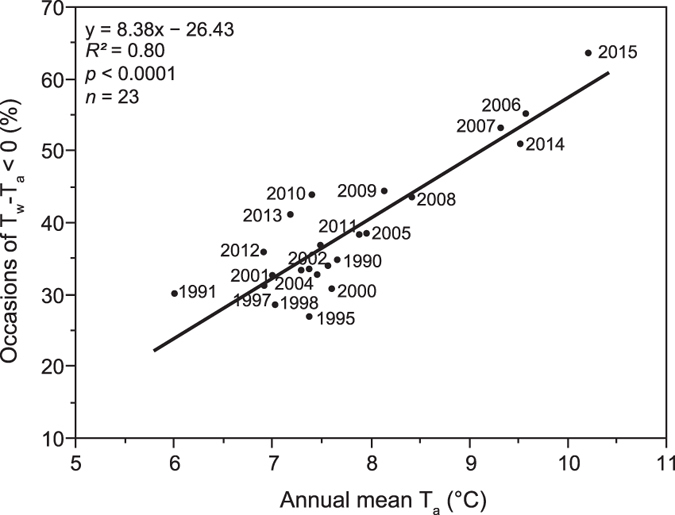
Increasing occasions of negative T_w_-T_a_ with increasing annual mean air temperatures. The percentage of negative T_w_-T_a_ is based on up to 8760 T_w_-T_a_ measurements (8784 T_w_-T_a_ measurements during leap years) that are available for 23 years from Lake Erken during 1989 to 2015. Shown is also a linear regression including the regression equation and regression statistics.

The term in square brackets, a meteorological forcing term, is not directly a function of *T*_*a*_. Because Γ is nearly linear with respect to *T*_*a*_ it is clear that the linear relationship between T_w_-T_a_ and T_a_ arises from the function Γ, *i.e.* through the process of surface evaporation. If this is the case, then the relationship should be detectable at all times and all places provided that radiative heat fluxes are comparable. Radiative heat fluxes usually show large diurnal and, outside the tropics, large seasonal cycles. To eliminate these variations we examined year-to-year variations in T_a_ and T_w_-T_a_ during a specific hour and a specific day of the year. Regardless of the day of the year or the hour of the day we always observed a strong negative relationship between T_a_ and T_w_-T_a_, based on data from Lake Erken (*p* < 0.0001 for 100 randomly chosen days and hours out of 8760 possible combinations; the relationship is graphically shown in Fig. 3 using Lake Erken data from the 21^st^ day of each month, both at midnight and at noon). Thus, T_w_-T_a_ and thereby Q_H_ becomes more negative at higher T_a_. Most critical are changes in the diurnal T_a_ cycle as these changes determine the frequency of upward and downward sensible heat fluxes. Using the longest available time series of year-round high-frequency temperature measurements from Lake Erken (Table 1) we found a strong increase in the occasions of negative T_w_-T_a_ with increasing annual mean T_a_ since 1989 (Fig. 4). During the coldest year (annual mean T_a_: 6 °C in 1991), T_w_-T_a_ reached negative values in 30% of more than 8500 T_w_-T_a_ measurements while T_w_-T_a_ reached negative values in as many as 64% during the warmest year (annual mean T_a_: 10 °C in 2015).

### Value of the citizen science project

When we initiated the citizen science project we were unaware of the very strong interest of school teachers to participate in research. The interest continued over the entire project. Some school classes were exceptionally well prepared when they asked their questions via Skype, while other classes provided inaccurate or unfilled protocols or sediment samples instead of water samples. Regardless, the enthusiasm of teachers and students was apparent in the evaluations. Overall, we consider the citizen science project a success. According to the teachers’ evaluations, the students increased their knowledge in natural sciences, and discovered a clear coupling between physics, mathematics, geography, chemistry and biology. For some students who had recently immigrated to Sweden, the experience was the first time that they had the opportunity to visit a remote lake in the Swedish landscape. According to the teachers, this was greatly beneficial. The teachers and students also thought that the media coverage of the students conducting field sampling was very positive. Throughout the project, there were intensive discussions among teachers within a Facebook group of more than 8500 Swedish natural science school teachers. This group was set up by a school teacher a few years ago and provides an excellent platform for this and future citizen science projects involving Swedish schools.

## Conclusion

We conclude that the citizen science project was highly successful and resulted in a win-win situation where (1) students increased their knowledge in natural sciences and their awareness of natural processes, and (2) scientists received valuable air and water temperature data. We also conclude that temperature measurements are suitable for a citizen science project as they are easy to perform, cheap, and based on our experience, reliable.

From the data which we received from the citizen scientists and from our own data, we can expect that ongoing and projected global warming will result in increasingly negative T_w_-T_a_, implying an increase in the downward sensible heat flux. This increase in downward sensible heat flux will enhance water column stability across inland water bodies, which will often result in reduced CO_2_ and CH_4_ transfer velocities towards the atmosphere. The global warming induced changes in these transfer velocities should be taken into consideration for future estimates of greenhouse gas emissions from inland waters, an important conclusion which we were able to derive from citizen science.

## Additional Information

**How to cite this article:** Weyhenmeyer, G. A. *et al*. Citizen science shows systematic changes in the temperature difference between air and inland waters with global warming. *Sci. Rep.* **7**, 43890; doi: 10.1038/srep43890 (2017).

**Publisher's note:** Springer Nature remains neutral with regard to jurisdictional claims in published maps and institutional affiliations.

## Figures and Tables

**Table 1:** Information on high-frequency temperature data automatically measured in lakes (at a water depth of ~0.5 m) and the overlying air (~1.5 m above the water) in 14 diverse lakes. The lakes are sorted from North to South.

Lake Country	Location of measuring site (Latitude and Longitude)	Lake surface area	Mean lake depth	Frequency of recorded measurements	Years of measurements considered in this study
Erken Sweden	59°50’20”N, 18°37’46”E	24.2 km^2^	9 m	Every 60 minutes	1989–2015
Peipsi Estonia/Russia	58°14’15”N, 27°28’28”E	3555 km^2^	7.1 m	Air temperature every 60 minutes, water temperature at 8 AM and 8 PM (local time)	2008–2015
Feeagh Ireland	53°55’12”N, 9°34’12”W	4.0 km^2^	14.5 m	Once daily	2013
Stechlin Germany	53°09’06”N, 13°10’34”E	4.3 km^2^	22.3 m	Every 60 minutes	2012– 2014
Bala UK	52°53’27”N, 3°37’12”W	4.1 km^2^	24 m	Every 60 minutes	2011
Buffalo Pound Canada	50°35'09”N, 105°23'02”W	24.4 km^2^	3.8 m	Every10 minutes	2015
Lake 239 Canada	49°39'48”N, 93°43'24”W	0.54 km^2^	11.0 m	Every 10 minutes	2012
Rimov Reservoir Czechia	48°50'56”N, 14°29'28”E	1.8 km^2^	15 m	Every 10 minutes ;	2015
Douglas US	45°33'54”N, 84°40'20”W	13.7 km^2^	8 m	Every 10 minutes	2011–2015
Harp Canada	45°22'48”N, 79°08'09”W	0.7 km^2^	13.3 m	Every 10 minutes	2013
Shelburne Pond US	44°23'38”N, 73°09'46”W	1.8 km^2^	3.4 m	Every 15 minutes	2015
Emerald US	36°35’49” N, 118°40’29”W	0.03 km^2^	6.0 m	Every 60 minutes	2011
Kivu Democratic Republic of the Congo/Rwanda*	1°43’30”S, 29°14’15”E	2700 km^2^	240 m	Every 30 minutes	2013
Curuai floodplain lake (Amazon) Brazil	2°04’12”S 55°03’58”W	2250 km^2^ at high water	6 m at high water	30 seconds for water temperature, every 5 minutes for air temperature	2014 (14 days of data)

^*^At Kivu Water temperature was monitored ~2 km southwest of the air temperature measurement site. Because the basin is spatially very homogeneous^9^, we considered this approach to provide reliable results.
